# Connected simultaneous rupture of the diaphragm and pericardium via congenitally fused site due to blunt trauma

**DOI:** 10.1186/s44215-022-00018-x

**Published:** 2023-02-02

**Authors:** Takashi Yamashita, Katsuyuki Asai, Hideto Ochiai, Toshikazu Kanai, Yuta Matsubayashi, Keizo Tanaka, Takashi Hashimoto

**Affiliations:** 1 General Thoracic Surgery, Hamamatsu Medical Center, 328, Tomitsuka, Nakaku, Hamamatsu, Shizuoka 432-8580 Japan; 2 Gastroenterological Surgery, Hamamatsu Medical Center, 328, Tomitsuka, Nakaku, Hamamatsu, Shizuoka 432-8580 Japan; 3 Cardiovascular Surgery, Hamamatsu Medical Center, 328, Tomitsuka, Nakaku, Hamamatsu, Shizuoka 432-8580 Japan

**Keywords:** Diaphragmatic rupture, Pericardial rupture, Blunt trauma, Pericardiodiaphragmatic hernia, Cardiac luxation, Emergency

## Abstract

**Background:**

In severe blunt trauma, multiple organ injuries are often observed. Patients with a ruptured diaphragm and pericardium are referred to as having pericardio-diaphragmatic rupture. However, few studies have reported a narrowly defined case of connected rupture of the diaphragm and pericardium via their congenitally fused site along with an abdominal visceral herniation and cardiac luxation into the thoracic cavity.

**Case presentation:**

A 78-year-old man presented to our hospital with left chest pain caused by a traffic accident. Contrast-enhanced computed tomography revealed a left diaphragmatic rupture and an intestinal herniation into the thoracic cavity. Surgical repair of the diaphragm was performed, and pericardial rupture was noted during surgery. It was considered that the laceration had spread via the congenitally fused site of the diaphragm and pericardium. The diaphragm was sutured, but the pericardium was left open because the laceration was large and the risk of cardiac incarceration was thought to be low. One year after the operation, no recurrence of diaphragmatic hernia was observed and any circulatory symptoms were not occurred.

**Conclusions:**

In cases of diaphragmatic laceration extending to the fused site of the pericardium, connected pericardial rupture should also be considered. It would be challenging to detect without intraoperative findings, and it is desirable to observe both the thoracic and abdominal cavities.

## Background

In severe blunt trauma, multiple organ injuries are often observed. Excessive pressure in the abdominal cavity can cause diaphragmatic rupture. Most cases occur on the left side because the liver offers some protection to the right side [1]. When the diaphragm ruptures, the abdominal viscera herniates into the thoracic cavity, a condition which is relatively easy to diagnose by taking radiological examinations [2]. Occasionally, the intestinal tract herniates into the pericardium; however, even in those cases, the air-fluid level can be observed in the pericardium; therefore, diagnosis should be possible. However, rupture of the pericardium alone is not easy to diagnose by radiological examinations [3]. If the diaphragmatic and pericardial rupture exist simultaneously, the diagnosis is even more difficult because of the complexed findings of radiological chest examinations. We encountered a rare case of connected simultaneous rupture of the diaphragm and pericardium, which appeared to be challenging to diagnose on radiological examinations and required exploration during surgery. Therefore, we report this rare case to prevent clinicians from overlooking pericardial rupture in cases of traumatic diaphragmatic rupture.

## Case presentation

A 78-year-old man presented to our hospital with left chest pain caused by a traffic accident. Three hours before emergency transport, he had driven a car and had a car accident that hit a wall at 40 km/h and then returned home by taxi. The chest pain persisted and worsened, and he was referred to the emergency department.

Upon arrival at the hospital, his vital signs demonstrated hypoxia (heart rate 79 beats per min, blood pressure 119/68 mmHg, respiratory rate 18, SpO_2_ 89% in ambient air). He was conscious (E4V5M6 in Glasgow coma scale) and could speak normally. He complained of left chest pain; however, no superficial wounds were detected. Electrocardiography showed incomplete right bundle branch brock, prolonged QTc interval, and no axial deviation; it was not seemed to be specific to any traumatic abnormalities.

**Fig. 1:**
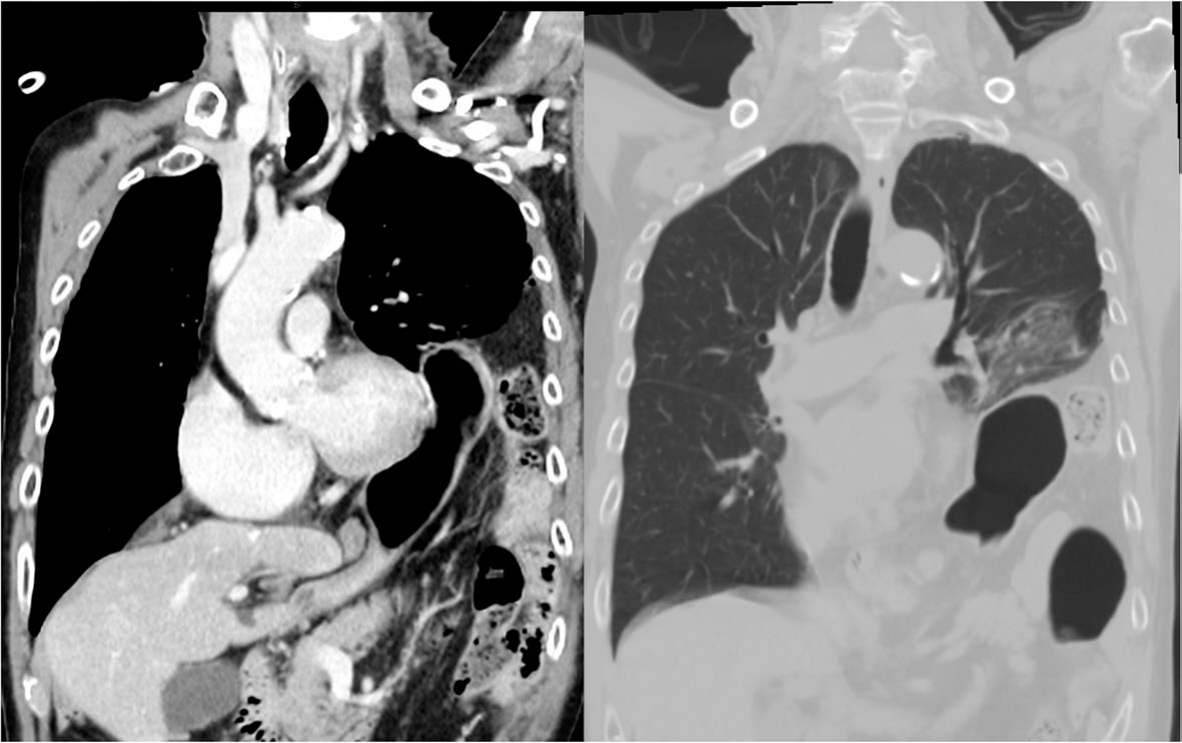
CT images at consultation. A herniation of the stomach, the small intestine, and the colon through into the left thoracic cavity and compressed lung was observed

Contrast-enhanced computed tomography (CT) revealed left diaphragm hernia, right traumatic lung cyst, sternum fracture, bilateral multiple rib fractures, 11th thoracic vertebral fracture, and multiple lumbar vertebral fractures. Herniation of the stomach, small intestine, and colon into the left thoracic cavity and compressed lung was observed, and no ischemic lesions were detected (Fig. 1). Emergency surgical repair was performed on the following day.

**Fig. 2:**
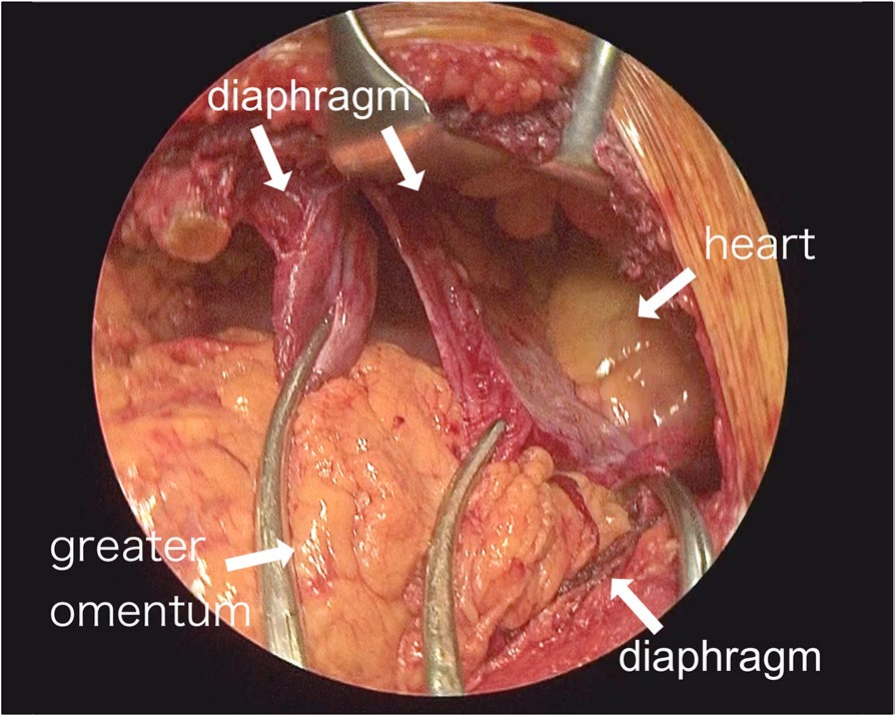
Intraoperative findings of diaphragm. The diaphragm having a T-shaped injury from the anterior to the lateral chest wall attachment part toward the apex of the diaphragm. The pericardium already ruptured retrospectively

**Fig. 3:**
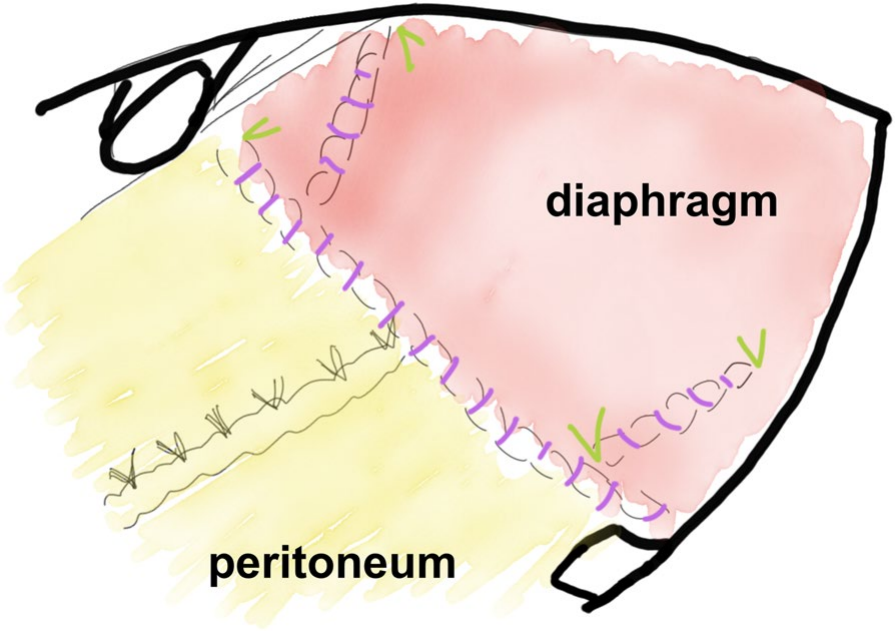
Operation schema. 2-0 synthetic non-absorbable braided thread (green) was used in a tensioned area and running suture by 2-0 synthetic absorbable thread (purple) was primarily used in combination

**Fig. 4:**
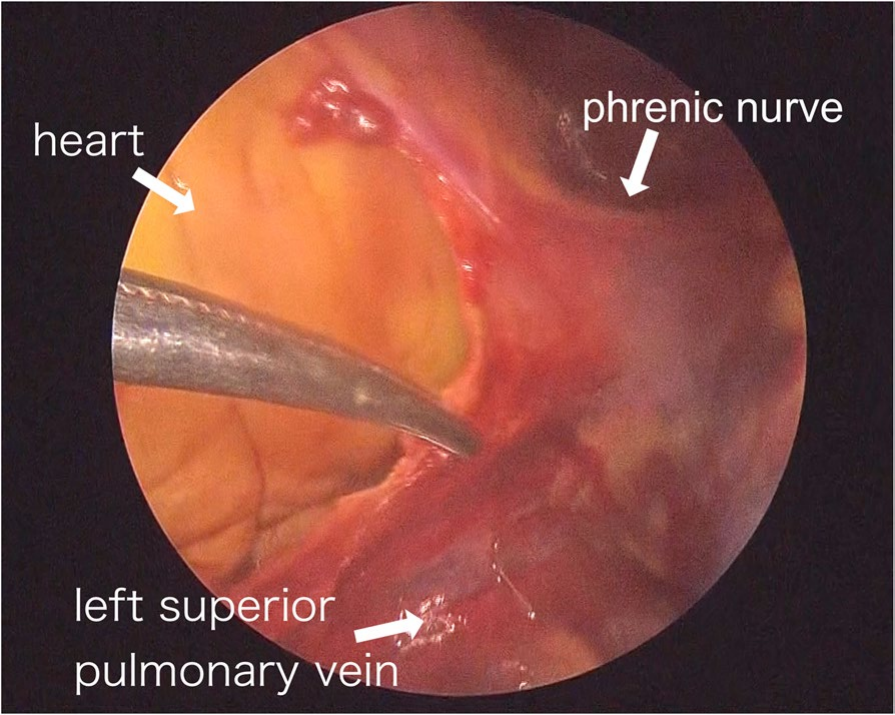
Intraoperative findings of pericardium. The laceration extending from the diaphragm to the hilar region on the dorsal side of the phrenic nerve

Although the repairs seemed to require a relatively large laparotomy, we hoped to minimize the invasiveness. Therefore, for the purpose of observing from inside the thoracic cavity, thoracoscopic exploration was performed in the right semi-lateral decubitus position under general anesthesia via port incision in the eighth intercostal space. The small intestine was observed in the thoracic cavity and a rupture of the left diaphragm was confirmed to ensure that it was not congenital. However, we switched to open thoracotomy in the eighth intercostal space with a 10-cm incision; the intraabdominal organs could not be returned easily. The incision was extended toward abdomen across the left costal arch, 20 cm thoraco-laparotomy, and exploration revealed that the diaphragm had a T-shaped injury from the anterior to the lateral chest wall attachment part toward the apex of the diaphragm (Fig. 2). The total length of diaphragm injury was 20 cm. After confirming that the herniated intestinal tract was undamaged, the diaphragm was sutured. While using 2-0 synthetic non-absorbable braided thread in a tensioned area, 2-0 synthetic absorbable thread was primarily used in combination (Fig. 3). After suturing the diaphragm, a 19Fr break drain was placed under the repaired diaphragm, and the peritoneum was closed first. Subsequently, when we attempted to place a 16 Fr thoracic drain in the thoracic cavity, we noticed that the heart was not covered with the pericardium. The diagnosis was pericardial rupture, rather than congenital pericardial defect, by confirming the edge of injured pericardium. The lacerations extended from the diaphragm to the hilar region on the dorsal side of the phrenic nerve (Fig. 4) and connected to the pericardio-diaphragmatic congenital fused site. Heart contusions were not observed. After some discussion, the pericardium remained intentionally unclosed because the large laceration was thought to have little risk of causing cardiac incarceration. Then the chest was closed. The total operating time was 3 h 18 min, and blood loss was 60 mL.

The postoperative period was stable, with no complications. Both drainage tubes were removed on post-operative day (POD) 5. On POD 12, the patient was infected with coronavirus disease due to the institutional cluster, and dexamethasone was required for worsening respiratory status. However, the patient recovered without any obvious sequelae and was discharged. One year after the operation, no recurrence of diaphragmatic hernia was observed and any circulatory symptoms were not occurred.

## Discussion

We report a rare case of the diaphragm and pericardium rupture in which the laceration had spread via the congenitally fused site of the diaphragm and pericardium, and an abdominal visceral hernia and cardiac luxation were observed. This case may be referred to as a case of pericardio-diaphragmatic rupture, which has become relatively commonly mentioned in the literature, although the word is actually refers to a disease with many different pathologies.

**Fig. 5:**
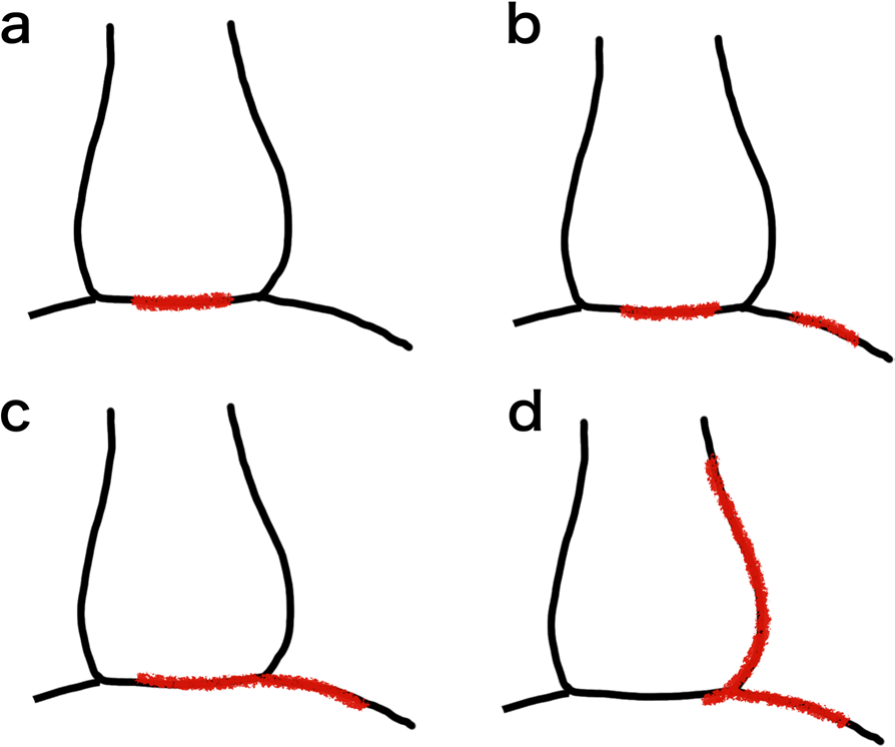
Ambiguity of the word “pericardio-diaphragmatic rupture.” The reported forms of pericardio-diaphragmatic rupture are as follows, **a** only the fused site is ruptured, **b** the fused site and the diaphragm are discontinuously damaged, and **c** the fused site laceration extends to the diaphragm. In our case, there was almost no damage to the fused site itself; therefore, it was considered that the main first injury was pericardium or diaphragm and spread to the another **d**

Diaphragmatic rupture from blunt thoracoabdominal trauma has been previously reported. Among those cases, abdominal viscera herniation into the pericardial cavity, or intrapericardial hernia, is relatively rare, and it is occasionally described as pericardio-diaphragmatic rupture because of a rupture of the congenitally fused diaphragmatic pericardium [4]. Additionally in this article, the cases of the fused site rupture with separated diaphragmatic rupture or the fused site rupture extending laterally into the diaphragm were described as pericardio-diaphragmatic ruptures [4]. Thus, the disease name “pericardio-diaphragmatic rupture” includes various pathological conditions, and our case does not have the same pathology as others that have been published (Fig. 5). Our case might also be expressed as pericardio-diaphragmatic rupture, which widely refers to injuries of the diaphragm and pericardium. However, narrowly defined cases of connected rupture of the diaphragm and pericardium with abdominal viscera herniation and cardiac luxation into the thoracic cavity have been scarcely reported. Moreover, there have been only two case reports of isolated delayed rupture of the diaphragm and pericardium [5, 6], whereas our case was a simultaneous rupture at the time of injury. Ours was a rare subtype of pericardio-diaphragmatic rupture.

However, so few studies have been published, pericardio-diaphragmatic rupture may not be a rare disease. The diagnosis of a large diaphragmatic rupture is relatively easy on CT [2] because of herniated abdominal viscera; however, pericardial rupture is difficult to diagnose even in cases in which CT scans are performed and needed a surgical exploration [3]. In our case, the co-occurrence of pericardial rupture along with diaphragmatic rupture was diagnosed during repair. The patient’s pericardial injury might have been present since the accident because his symptoms and hemodynamics did not change significantly after admission; however, it could not be diagnosed preoperatively. Although we could not determine which one was damaged first, the diaphragm and pericardium were fused congenitally [7], it was considered that the laceration had spread via the congenitally fused site in this case. Therefore, we suggest that connected pericardial rupture should be considered in cases of diaphragmatic laceration extending to the congenitally fused pericardial site. Additionally, as previously reported, pericardial rupture may not be found during laparotomic repair [8]. In this case, pericardial rupture did not lead to a life-threatening condition because the abdominal viscera did not compress his heart in a closed pericardium. However, cardiac luxation caused by pericardial rupture can be sometimes fatal [9], and it is important to know whether it is present. Definitive diagnosis of pericardial rupture is considered challenging without intrathoracic surgical exploration; therefore, it is better to explore both the thoracic and abdominal cavities during surgical repair of traumatic diaphragmatic rupture in some uncertain situations. In that sense, it is important to determine whether the congenitally fused site is damaged.

In our case, the pericardial rupture was not closed because the large laceration was thought to have little risk of causing cardiac incarceration. Whether and how to close the pericardium after cardiac dislocation remains controversial, even in elective operations [10]. However, as in similar cases, we recommend that artificial materials should not be used for pericardial closure when diaphragmatic hernia is complicated. In the acute setting, occult damage and subsequent infectious complications should be considered, even if intestinal damage is not observed during the operation. If the pericardium or diaphragm must be repaired, autologous fascia lata is a better option because of its higher resistance to infection than artificial materials [11, 12]. In any case, since it is necessary to confirm that pericardium is injured or not for subsequent treatments, it is desirable to observe both the thoracic and abdominal cavities by surgical procedures.

## Data Availability

Not applicable.

## Abbreviations

CT: Computed tomography
POD: Postoperative day

## References

[1] Hanna WC, Ferri LE, Fata P, Razek T, Mulder DS. The current status of traumatic diaphragmatic injury: lessons learned from 105 patients over 13 years. Ann Thorac Surg. 2008;85:1044–8. 18291194 10.1016/j.athoracsur.2007.10.084

[2] Magu S, Agarwal S, Singla S. Computed tomography in the evaluation of diaphragmatic hernia following blunt trauma. Indian J Surg. 2012;74:288–93. 23904715 10.1007/s12262-011-0390-7 PMC3444616

[3] Graef F, Walter S, Baur A, Tsitsilonis S, Moroder P, Kempfert J, et al. Traumatic cardiac dislocation - a case report and review of the literature including a new classification system. J Trauma Acute Care Surg. 2019;87:944–53. 31453985 10.1097/TA.0000000000002445

[4] Sharma OP. Pericardio-diaphragmatic rupture: five new cases and literature review. J Emerg Med. 1999;17:963–8. 10595880 10.1016/s0736-4679(99)00124-9

[5] Kamiyoshihara M, Nagashima T, Ibe T, Takeyoshi I. Rupture of the diaphragm and pericardium with cardiac herniation after blunt chest trauma. Gen Thorac Cardiovasc Surg. 2010;58:291–4. 20549460 10.1007/s11748-009-0520-3

[6] Gunn JM, Savola J, Isotalo K. Left-sided diaphragmatic and pericardial ruptures with subluxation of the heart after blunt trauma. Ann Thorac Surg. 2012;93:317–9. 22186460 10.1016/j.athoracsur.2011.05.046

[7] van Tornout F, van Leuven M, Parry W. Pericardio-diaphragmatic avulsion and concomitant rupture of the central tendon of the diaphragm. Eur J Cardiothorac Surg. 2004;26:655–7. 15302069 10.1016/j.ejcts.2004.05.038

[8] Witkowski Z, Lasek J, Wujtewicz M, Stasiak M, Marks W, Kawecka A. Pericardiodiaphragmatic rupture and cardiac herniation after multiple blunt trauma: diagnostic and therapeutic difficulties. J Thorac Cardiovasc Surg. 2005;130:e1. 10.1016/j.jtcvs.2005.07.054 16307985

[9] Fulda G, Brathwaite CE, Rodriguez A, Turney SZ, Dunham CM, Cowley RA. Blunt traumatic rupture of the heart and pericardium: a ten-year experience (1979-1989). J Trauma. 1991;31:167–72 discussion 172-3. 1994075

[10] Boyd WD, Tyberg J, v., Cox JL. A review of the current status of pericardial closure following cardiac surgery. Expert Rev Cardiovasc Ther. 2012;10:1109–18. 23098147 10.1586/erc.12.87

[11] Yamashita T, Asai K, Suzuki K. Reconstructed diaphragm by fascia lata: 13 years in vivo. Ann Thorac Surg. 2021;111:e247–8. 32956673 10.1016/j.athoracsur.2020.06.127

[12] Kobayashi H, Nomori H, Mori T, Shibata H, Yoshimoto K, Ohba Y. Extrapleural pneumonectomy with reconstruction of diaphragm and pericardium using autologous materials. Ann Thorac Surg. 2009;87:1630–2. 19379936 10.1016/j.athoracsur.2008.09.068

